# IL-34 Induces the Differentiation of Human Monocytes into Immunosuppressive Macrophages. Antagonistic Effects of GM-CSF and IFNγ

**DOI:** 10.1371/journal.pone.0056045

**Published:** 2013-02-08

**Authors:** Etienne D. Foucher, Simon Blanchard, Laurence Preisser, Erwan Garo, Norbert Ifrah, Philippe Guardiola, Yves Delneste, Pascale Jeannin

**Affiliations:** 1 LUNAM Université, Université d'Angers, Angers, France; 2 Inserm, unit 892, Angers, France; 3 CNRS, unit 6299, Angers, France; 4 Université d'Angers, Centre Hospitalier Universitaire, Laboratoire d'Immunologie et d'Allergologie, Angers, France; 5 Université d'Angers, Centre Hospitalier Universitaire, Service des Maladies du Sang, Angers, France; 6 Plateforme SNP, Transcriptome & Epigénomique, Centre Hospitalier Universitaire, Angers, France; University of Lyon, France

## Abstract

IL-34 is a recently identified cytokine that signals via the M-CSF receptor and promotes monocyte survival. Depending on the environment, monocytes can differentiate into macrophages (Mφ) or dendritic cells (DC). A wide spectrum of Mφ and DC subsets, with distinct phenotypes and functions, has been described. To date, the phenotype of monocytes exposed to IL-34 remains unexplored. We report here that IL-34 induces the differentiation of monocytes into CD14^high^ CD163^high^ CD1a^−^ Mφ (IL-34-Mφ). Upon LPS stimulation, IL-34-Mφ exhibit an IL-10^high^ IL-12^low^ M2 profile and express low levels of the costimulatory molecules CD80 and CD86. IL-34-Mφ exhibit poor T cell costimulatory properties, and have potent immunosuppressive properties (decrease of TCR-stimulated T cell proliferation). For all the parameters analyzed, IL-34-Mφ are phenotypically and functionally similar to M-CSF-Mφ. IL-34 appears as efficient as M-CSF in inducing the generation of immunosuppressive Mφ. Moreover, the generation of IL-34-Mφ is mediated through the M-CSF receptor, is independent of endogenous M-CSF consumption and is potentiated by IL-6. In an attempt to identify strategies to prevent a deleterious M2 cell accumulation in some pathological situations, we observed that IFNγ and GM-CSF prevent the generation of immunosuppressive Mφ induced by IL-34. IFNγ also switches established IL-34-Mφ into immunostimulatory Mφ. In conclusion, we demonstrate that IL-34 drives the differentiation of monocytes into immunosuppressive M2, in a manner similar to M-CSF, and that IFNγ and GM-CSF prevent this effect.

## Introduction

Circulating monocytes are precursors that exhibit some effector functions [1], [2]. They can differentiate into a variety of tissue-resident macrophages (Mφ), dendritic cells (DC), and osteoclasts [3]. Macrophages play a central role in tissue homeostasis, host defense, inflammation, and tissue repair. They also participate to tissue remodeling in ontogenesis and to the control of metabolic functions [4]. Macrophages, characterized by their plasticity and heterogeneity [4], [5], [6], are highly sensitive to their environment and, according to the signals encountered (microorganisms, damaged self, cytokines), may acquire distinct functional phenotypes and exhibit opposite activities (i.e. pro- versus anti-inflammatory, immunosuppressive versus immunostimulatory) [4]. Pathophysiological situations are usually associated with changes in Mφ phenotypes and functions and, in some of them, Mφ with different phenotypes coexist [4].

Reflecting the Th1/Th2 dichotomy, two states of Mφ polarization, called M1 and M2, which represent extremes of a spectrum of activation states, have been described [6]. The M1 phenotype results from the stimulation of Mφ with interferon-γ (IFNγ), alone or with microbial products (mainly Toll-like receptor [TLR] agonists) or cytokines (such as tumor necrosis factor-α [TNFα]). M1 cells produce high levels of IL-12 (IL-12^high^) and low levels of IL-10 (IL-10^low^), proinflammatory cytokines, nitrogen and oxygen intermediates, and have potent microbicidal and tumoricidal activities [4], [7], [8]. M2 cells, also called “alternatively activated macrophages”, were initially described as cells generated in the presence of IL-4 or IL-13. M2, characterized by an IL-12^low^ IL-10^high^ phenotype, have potent phagocytic properties and are involved in tissue remodeling, tumor progression, and immune suppression. Other subsets of IL-10^high^ IL-12^low^ M2 cells (called M2-like), that differ phenotypically from IL-4-Mφ, have also been characterized [6], [7], [9], [10]. However, this classification does not reflect the extreme plasticity of Mφ and the continuum of Mφ subsets (from M1 to M2) that can exhibit characteristics shared by more than one Mφ population. Another classification has thus been proposed, based on three different homeostatic activities: classically activated Mφ (involved in host defence and antitumoral immunity), wound healing Mφ (production of extracellular matrix components) and regulatory Mφ (immune regulation and resolution of inflammation) [11].

IL-34 was identified by functional screening of a library of secreted proteins, based on its ability to support human monocyte survival and to promote, with the same efficiency as macrophage colony-stimulating factor (M-CSF), the formation of the colony-forming unit-macrophage (CFU-M) in human bone marrow cell cultures [12]. Murine IL-34 also promotes peritoneal Mφ survival and bone marrow progenitor cell proliferation [13]. IL-34 is a dimeric glycoprotein which presents no amino-acid sequence homology with M-CSF, although human IL-34 secondary structure prediction suggests the existence of four α-helical bundles, as observed for human M-CSF [14], [15], [16]. The M-CSF receptor (M-CSF-R), also called colony-stimulating factor 1-receptor, c-fms or CD115, has been identified as an IL-34 receptor [12]. IL-34 mRNA is expressed in various tissues and is most abundant in the spleen [12]. The spatiotemporal expression patterns of IL-34 and M-CSF mRNA differ, suggesting that IL-34 and M-CSF may have non-redundant and complementary roles [17], [18]. In agreement with these observations, two recent studies have evidenced the role of IL-34 in the homeostasis of Langherans cells and microglia [19], [20].

When used in combination with receptor activator of nuclear factor kappa-B ligand (RANKL), IL-34 can replace M-CSF for human and murine osteoclast differentiation [21], [22]. In vivo, administration of IL-34 reduces bone mass [21], [22] and the expression of IL-34 in an M-CSF-specific manner rescues osteopetrotic deficiencies in M-CSF-deficient mice [17]. However, studies suggest that IL-34 and M-CSF may also have non-overlapping properties [12]. IL-34 and M-CSF differ in their ability to induce the production of the chemokines MCP-1 and eotaxin by human monocytes [13]. They also differently affect the migration of a murine myeloid cell line [13], while their ability to promote the survival of murine Mφ or the growth of murine bone marrow progenitors is identical [13]. In support, it has been suggested that IL-34 and M-CSF may interact with distinct regions of M-CSF-R and activate different signaling pathways [13], [16]. The potential physiopathological roles of IL-34 remain poorly described. IL-34 has been reported overexpressed in rheumatoid arthritis [23] and involved in RA-associated osteoclastogenesis [24]. In breast cancer, some antitumoral drugs induce the production of both IL-34 and M-CSF by mammary epithelial cells, which may favor the recruitment of Mφ into the tumor microenvironment [25].

Taken into account the ability of IL-34 to promote human monocyte survival and the central role played by Mφ in physiological and pathological situations, the objective of this study was to analyze the phenotype and function of human Mφ generated in the presence of IL-34. We report that IL-34 gives rise to immunosuppressive Mφ with an IL-10^high^ IL-12^low^ phenotype and demonstrate that granulocyte-macrophage colony-stimulating factor (GM-CSF) and IFNγ prevent their generation, while IL-6 pushes them toward a more pronounced phenotype.

## Materials and Methods

### Ethics Statement

PBMCs were obtained from healthy human volunteers (Blood collection center, Angers, France; agreement #ANG-2003-2).

### Cytokines

IL-4 and GM-CSF were purchased from CellGenix (Freiburg, Germany). IL-3, IL-6, IL-7, IL-8, IL-9, IL-10, IL-12, IL-15, IL-21, and IFNγ were from Immunotools (Friesoythe, Germany), IL-1β, IL-2, and OSM were from Miltenyi Biotec (Bergisch Gladbach, Germany). IL-5, IL-17A, IL-17E, IL-19, IL-20, IL-22, IL-23, IL-24, IL-26, IL-29, IL-34 and TNFα were from R&D Systems (Abingdon, United Kingdom). M-CSF was from ORF Genetics (Kopavogur, Iceland).

### Cell purification and culture

Peripheral blood mononuclear cells (PBMC) were isolated with Lymphocyte Separation Medium (PAA Laboratories, Pashing, Austria). CD14^+^ monocytes and CD4^+^ T cells were sorted from PBMC by positive magnetic sorting (Miltenyi Biotec). CD4^+^ memory T cells were sorted from PBMC by negative depletion followed by a positive magnetic sorting of CD45RO^+^ T cells (both from Miltenyi Biotec). Myeloid cells were cultured in medium consisting of RPMI 1640 medium (Lonza, Verviers, Belgium) supplemented with 10% fetal calf serum (PAA Laboratories), 2 mM l-glutamine, 1 mM sodium pyruvate, 0.1 mM non essential amino acids, 10 mM Hepes, 100 U/ml penicillin and 100 µg/ml streptomycin (all from Lonza). CD4^+^ T cells were cultured in serum-free X-VIVO-20 medium (Lonza).

### Macrophage subset generation

Macrophage subtypes are referred to as the cytokines used for their generation. IL-34-Mφ, M-CSF-Mφ, and GM-CSF-Mφ were generated by exposing monocytes (1×10^6^ cells/ml) for 5 days to 50 ng/ml IL-34, 50 ng/ml M-CSF or 20 ng/ml GM-CSF, respectively. In some experiments, monocytes were cultured with 50 ng/ml IL-34 plus 50 ng/ml IL-6. To generate the IL-4-Mφ, IL-1β-Mφ or IL-10-Mφ subsets, monocytes were first exposed for 3 days to 20 ng/ml GM-CSF and then for 2 days to 50 ng/ml IL-4, IL-1β or IL-10 [6]. M-CSF+IL-6-Mφ were generated by exposing monocytes to 50 ng/ml M-CSF plus 50 ng/ml IL-6 [10]. In some experiments, day-5 Mφ were activated for 48 h with 200 ng/ml lipopolysaccharide (LPS) (from *E. coli* serotype O111:B4; Sigma-Aldrich, St Louis, MO).

### Cytokine quantification

IL-10 and IL-12 were quantified using commercial ELISA from Diaclone (Dijon, France) and IL-34 using a commercial ELISA from R&D Systems.

### Flow cytometry analysis

Cells were incubated with FITC-labeled anti-CD80, PE-labeled anti-CD86, APC-labeled anti-CD4 (all from BD Pharmingen, San Diego, CA), PE-labeled anti-CD14 (Dako, Glostrup, Denmark), PC5-labeled anti-ILT3 (Beckman Coulter, Marseille, France), APC-labeled anti-CD163 or PE-labeled anti-CD206 (R&D Systems) mAbs. Data were acquired using a FACScalibur flow cytometer (BD Biosciences, San Jose, CA) and analyzed with the FlowJo software (Tree Star, Ashland, OR). Results are expressed as mean fluorescence intensities (MFI) after subtraction of the value obtained with the isotype control mAb or as a percentage of inhibition determined as follows: (A–B)/A×100, where A and B are the MFI values in the absence or presence of the inhibitor, respectively.

### Cell viability analysis

The viability of cells was evaluated by annexin V labeling. Data were acquired using a FACScalibur and analyzed with the FlowJo software. Results are expressed as a percentage of annexin V^−^ viable cells.

### Analysis of chemokine and chemokine receptor mRNA expression

IL-34-Mφ, M-CSF-Mφ and GM-CSF-Mφ were stimulated or not for 6 h with 200 ng/ml LPS. After cell lysis using the Trizol reagent (Life technologies, Saint Aubin, France), total RNA were extracted using the RNeasy Micro kit according to the manufacturer recommendation (Qiagen, Hilden, Germany) and reverse transcribed using the Superscript II reverse transcriptase (Life technologies). The expression of CCL1, CCL2, CCL5, CCL18, CCL17, CCL22, CCL24, CXCL9, CXCL10, CXCL11, CXCL16, CX3CL1, CCR1, CCR2, CCR8, CX3CR1, CXCR1, and CXCR2 mRNA was analyzed by qPCR using the iQ SYBR Green Supermix (Biorad, Marnes-la-Coquette, France); primer sequences are available upon request. Specific gene expression was calculated using the 2^−ΔΔCT^ method using GAPDH as calibrator. The transcriptional profiles were evaluated in four donors. Omics Explorer 2.3 software was used for hierarchical clustering and principal-component analysis (PCA) (Qlucore, Lund, Sweden). Hierarchical clustering of both samples and transcripts was performed using average linkage method and the Euclidean metric, with each variable being normalized to mean 0 and variance 1.

### Inhibition of T cell proliferation and mixed lymphocyte reaction

In T cell proliferation assays, 10^5^ allogeneic CD4^+^ CD45RO^+^ memory T cells, previously incubated with 3 µM CFDA-SE (Molecular Probes, Carlsbad, CA), were cultured in X-VIVO-20 medium supplemented with 20 U/ml IL-2, in 96-well flat-bottomed plates, previously coated with 1 µg/ml anti-CD3 mAb (clone OKT3; ATCC, Manassas, VA), without or with 2×10^4^ allogeneic LPS-activated Mφ subsets. In MLR experiments, 10^5^ CFDA-SE stained CD4^+^ T cells were cultured, as described above, in non-coated plates without or with 2×10^4^ allogeneic LPS-activated Mφ subsets, in the presence of 20 U/ml IL-2. At day 7, cells were stained with APC-labeled anti-CD4 mAb for gating on T cells and the proliferation of CD4^+^ T cells was measured by analyzing CFDA-SE dilution. Data were subsequently analyzed using the FlowJo Proliferation Platform software v7.6.5 (Tree Star, Ashland, OR).

### Inhibition and reversion of macrophage polarization

In order to evaluate the role of CD15 and/or M-CSF in the generation of IL-34-Mφ, M-CSF-Mφ and GM-CSF-Mφ were generated in the presence or absence of 4 µg/ml anti-M-CSF or isotype control mAbs (both from R&D Systems), or 1 µM GW2580 (LC Laboratories, Woburn, MA), a CD115 tyrosine kinase inhibitor. In order to evaluate the capacity of some cytokines to prevent the generation of IL-34-Mφ, 50 ng/ml IL-1β, IL-2, OSM, IL-4, GM-CSF, IL-3, IL-7, IL-8, IL-9, IL-12, IL-15, IL-21, IFNγ, IL-5, IL-17A, IL-17E, IL-19, IL-20, IL-22, IL-23, IL-24, IL-26, IL-29, or TNFα were added to monocytes at day 0 together with IL-34. In reversion experiments, established day-5 IL-34-Mφ were incubated with 50 ng/ml GM-CSF or IFNγ for 3 days. In all experiments, Mφ were stimulated for 48 h with 200 ng/ml LPS before quantification of IL-12 and IL-10 by ELISA and analysis of CD80 and CD86 expression by flow cytometry.

### Statistical analysis

Data are shown as mean ± SD and were analyzed by the Mann-Whitney test, with p<0.05 being considered significant.

## Results

### IL-34 induces the differentiation of human monocytes into macrophages

**Figure 1:**
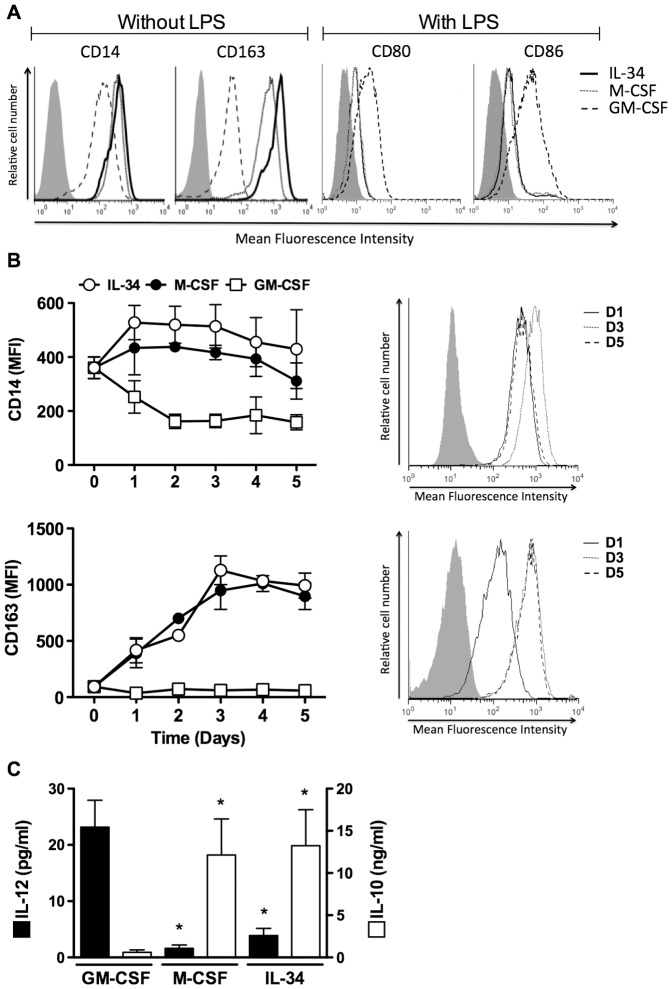
IL-34 induces monocyte differentiation into M2 cells. *A, IL-34-M*φ *exhibit a CD14^high^ CD163 ^high^ CD80^low^ CD86^low^ phenotype.* The expression of CD14 and CD163 (before LPS stimulation) and of CD80 and CD86 (after LPS stimulation) was analyzed by flow cytometry on IL-34-Mφ, M-CSF-Mφ, and GM-CSF-Mφ. For each marker, the grey histogram corresponds to isotype control mAb and is similar in the 3 experimental conditions. Results are representative of 1 of 5 experiments. *B, IL-34-Mφ polarization results in the maintenance of CD14 and in the acquisition of CD163.* The expression of CD14 and CD163 was analyzed by flow cytometry during the 5-day polarization of IL-34-Mφ, M-CSF-Mφ, and GM-CSF-Mφ (left panels). Right panels, flow cytometry histograms of CD14 and CD163 expression by monocytes cultured with IL-34 and analyzed at day 1, day 3, and day5. *C, IL-34-Mφ display an IL-10^high^ IL-12^low^ phenotype.* IL-10 and IL-12 were quantified in the supernatants of IL-34-Mφ, M-CSF-Mφ, and GM-CSF-Mφ after LPS stimulation. Results are expressed in pg/ml (IL-12) or ng/ml (IL-10) (mean ± SD, n = 5). * p<0.05, compared to GM-CSF.

In order to investigate the role of IL-34 on human monocyte differentiation, highly purified peripheral blood monocytes were cultured with IL-34 for 5 days. Preliminary experiments showed, as previously reported [12], [13], that IL-34 promotes monocyte survival; in our experimental conditions, the maximal survival was obtained with a concentration of 50 ng/ml IL-34 (data not shown). The phenotype of cells cultured with IL-34 was analyzed by flow cytometry and their ability to produce IL-10 and IL-12 was assessed by ELISA. IL-34 induced monocyte differentiation into cells exhibiting Mφ characteristics, as evidenced by the expression of CD14 and CD163 (Fig. 1A), but not dendritic cell features (absence of CD1a and no induction of CD83 upon LPS stimulation; data not shown). These differentiated cells were called IL-34-Mφ. As controls, the macrophage differentiation factors GM-CSF (GM-CSF-Mφ) and M-CSF (M-CSF-Mφ) also gave rise to CD1a^−^ CD14^+^ CD163^+^ cells (Fig. 1A and data not shown) [7], [10], [26]. IL-34-Mφ and, as previously reported [10], M-CSF-Mφ, expressed higher levels of CD14 (2.5-fold increase) and CD163 (25-fold increase) than GM-CSF-Mφ (Fig. 1A). IL-34 and M-CSF were equivalent in maintaining the level of CD14 (Fig. 1B, left panel) and in increasing the expression of CD163 (Fig. 1B, right panel) during the 5-days differentiation process. Interestingly, the induction of CD163 and the expression of CD14 were homogenous on the whole cell population (Fig. 1B), suggesting that IL-34 and M-CSF induce the differentiation of monocytes into a Mφ subset. In contrast, GM-CSF decreased the expression of CD14 and did not induce the expression of CD163 (Fig. 1B, right panels). Finally, the expression of MHC-I, MHC-II, CD54, CD80, CD86, B7-H1, ILT3 and CD206 was similar on IL-34-Mφ and M-CSF-Mφ and was different to the one exhibited by GM-CSF-Mφ (Table 1).

**Table 1:** Analysis of cell surface markers on macrophage subsets.

	Without LPS			With LPS		
Markers	IL-34-Mφ	M-CSF-Mφ	GM-CSF-Mφ	IL-34-Mφ	M-CSF-Mφ	GM-CSF-Mφ
CD14	++++	++++	+++	+++	+++	++++
CD163	+++++	+++++	++	+++++	+++++	+++
CD80	−	−	+	−	−	+
CD86	+	+	+	+	+	++
B7H1	−	−	−	+	+	+
ILT3	++++	++++	+++	++	++	+
CD54	+	+	++	++	+++	+++
MHC 1	+++	+++	+++	+++	+++	++++
MHC 2	++++	++++	+++	+++	+++	++++
CD206	−	−	+++	+	+	++

The expression of the indicated markers was analyzed by FACS on IL-34-Mφ, M-CSF-Mφ and GM-CSF-Mφ, stimulated or not for 48 h with 200 ng/ml LPS. Results are expressed as mean fluorescence intensity values (after subtraction of the MFI obtained with the isotype control mAb) and are representative of 4 separate experiments. +++++ indicates MFI greater than 1000; ++++, MFI greater than 400 and less than 1000; +++, MFI greater than 200 and less than 400; ++, MFI greater than 100 and less than 200; +, MFI greater than 20 and less than 100 and, − indicates MFI less than 20.

These results show that IL-34 supports the differentiation of human peripheral blood monocytes into Mφ.

### IL-34-Mφ exhibit a M2 phenotype

We then analyzed the phenotype of activated IL-34-Mφ. Upon LPS stimulation, IL-34-Mφ and, as expected [7], M-CSF-Mφ, produced weak or undetectable levels of IL-12 (Fig. 1C). In contrast, GM-CSF-Mφ produced higher levels of IL-12 and lower levels of IL-10 than IL-34-Mφ and M-CSF-Mφ (Fig. 1C). This IL-10^high^ IL-12^low^ phenotype of IL-34-Mφ is typical of a M2 polarization [4], [6], [7], [9]. The expression of CD80, CD86 (Fig. 1A) and CD206 (Table 1) was upregulated on LPS-stimulated GM-CSF-Mφ, compared to LPS-stimulated IL-34-Mφ and M-CSF-Mφ. In contrast, the expression of MHC-I, MHC-II, CD54, B7-H1 and ILT3 was similar on LPS-stimulated IL-34-Mφ, M-CSF-Mφ and GM-CSF-Mφ (Table 1).

**Figure 2:**
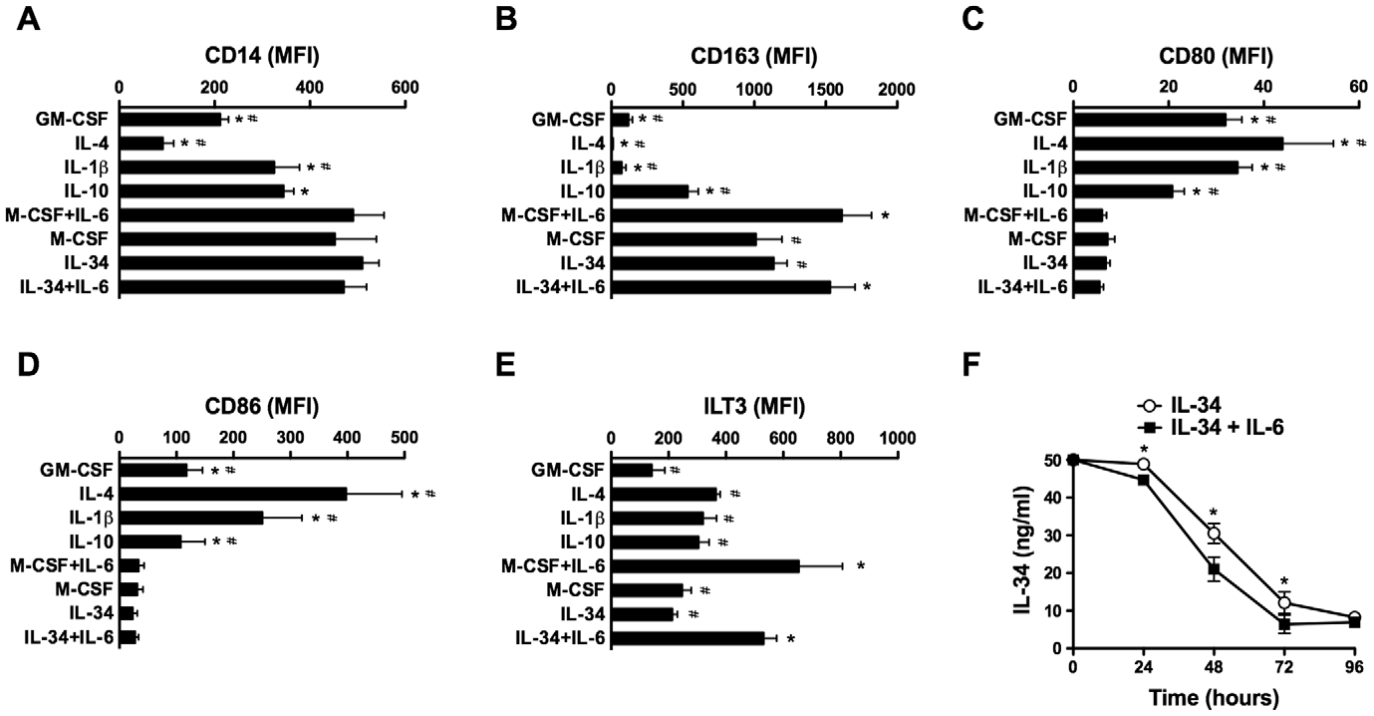
IL-34 induces M2 cell generation. *A–E, IL-34 induces monocyte differentiation into M2*. Macrophage subsets were generated as described in the Material & Methods section. The expression of CD14 (*A*) and CD163 (*B*) was analyzed by flow cytometry on non stimulated cells. The expression of CD80 (*C*), CD86 (*D*) and ILT3 (*E*) was analyzed by flow cytometry after LPS stimulation. Results are expressed in MFI values (mean ± SD, n = 5). * p<0.05, compared to IL-34; # p<0.05, compared to IL-34+IL-6. *F, IL-6 favors IL-34 consumption*. IL-34 was quantified at different time-points in the supernatants of monocytes exposed to 50 ng/ml IL-34, without or with 50 ng/ml IL-6. Results are expressed in ng/ml (mean ± SD, n = 4). * p<0.05.

Four M2 cell subsets elicited by exposing GM-CSF-Mφ to IL-4 (IL-4-Mφ), IL-1β (IL-1β-Mφ), IL-10 (IL-10-Mφ) [4], [5], [6], [27] or by exposing monocytes to M-CSF plus IL-6 (M-CSF+IL-6-Mφ) [10], have been described. These subsets are. The CD14^high^ CD163^high^ CD80^low^ CD86^low^ phenotype of IL-34-Mφ is similar to the one of M-CSF-Mφ and differs from IL-4-, IL-1β- and IL-10-Mφ (Fig. 2A–D).

We have previously demonstrated that IL-6 favors M-CSF consumption by monocytes and, in the presence of M-CSF, gives rise to cells with a more pronounced M2 phenotype [10], [28]. Similarly, cells cultured with IL-34 and IL-6 (called IL-34+IL-6-Mφ) express higher levels of CD163 (Fig. 2B) and ILT3 (Fig. 2E) than IL-34-Mφ. Interestingly, the concentrations of IL-34 were reduced in the supernatants of cells cultured with IL-34 plus IL-6 compared to cells cultured with IL-34 alone (Fig. 2F). This results, added to the fact that no IL-34 mRNA was detected during the 5-days culture with IL-34, in the absence or presence of IL-6 (data not shown), suggests that IL-6 enhances the consumption of IL-34 during the differentiation process.

**Figure 3:**
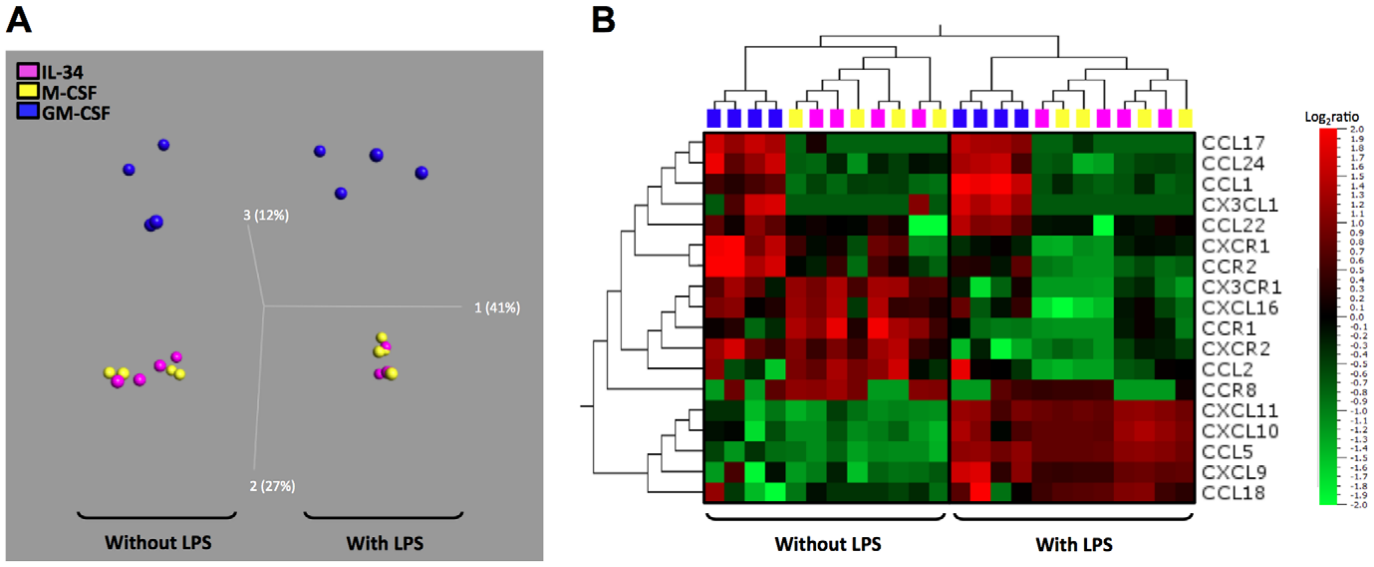
Analysis of chemokine and chemokine receptor mRNA expression by macrophages. *A Three-dimensional projection of PCA of IL-34-Mφ, M-CSF-Mφ and GM-CSF-Mφ*. Each dot represents one Mφ subset of one donor, stimulated or not for 6 h with LPS, based on values of all 18 studied chemokines/chemokine receptors. The percentage of variances is depicted on the three axes. *B,Two-dimensional unsupervised hierarchical clustering analysis of the chemokines/chemokine receptors expression in the Mφ subset*. The normalized values for each chemokine/chemokine receptors from four different donors is depicted according to the color scale, where red and green represent expression above and below the mean, respectively.

Previous studies suggested that M1 and M2 exhibit different chemokine and chemokine receptor patterns [6], [29], [30], [31]. PCA of the transcriptional profiles evidenced a relationship between samples, according to the polarization, and revealed the absence of segregation between IL-34-Mφ and M-CSF-Mφ (Fig. 3A). Moreover, IL-34-Mφ and M-CSF-Mφ have lower levels of the mRNA encoding CCL1, CCL17, CCL22, CCL24 and CX3CL1 than GM-CSF-Mφ (Fig. 3B). We also observed that the transcripts encoding the chemokine receptors CCR2 and CXCR1 were lower in IL-34-Mφ and M-CSF-Mφ than in GM-CSF-Mφ (Fig. 3B). Stimulation of the Mφ with LPS did not modify this dual expression (Fig. 3). The expression of the mRNA encoding the other chemokines and chemokine receptors were equivalent in the three Mφ subsets (Fig. 3B).

Together, these results show that IL-34-Mφ exhibit a CD14^high^ CD163^high^ CD80^low^ CD86^low^ M2 phenotype and that IL-6 pushes them into cells with a more pronounced phenotype.

### IL-34-Mφ are immunosuppressive

**Figure 4:**
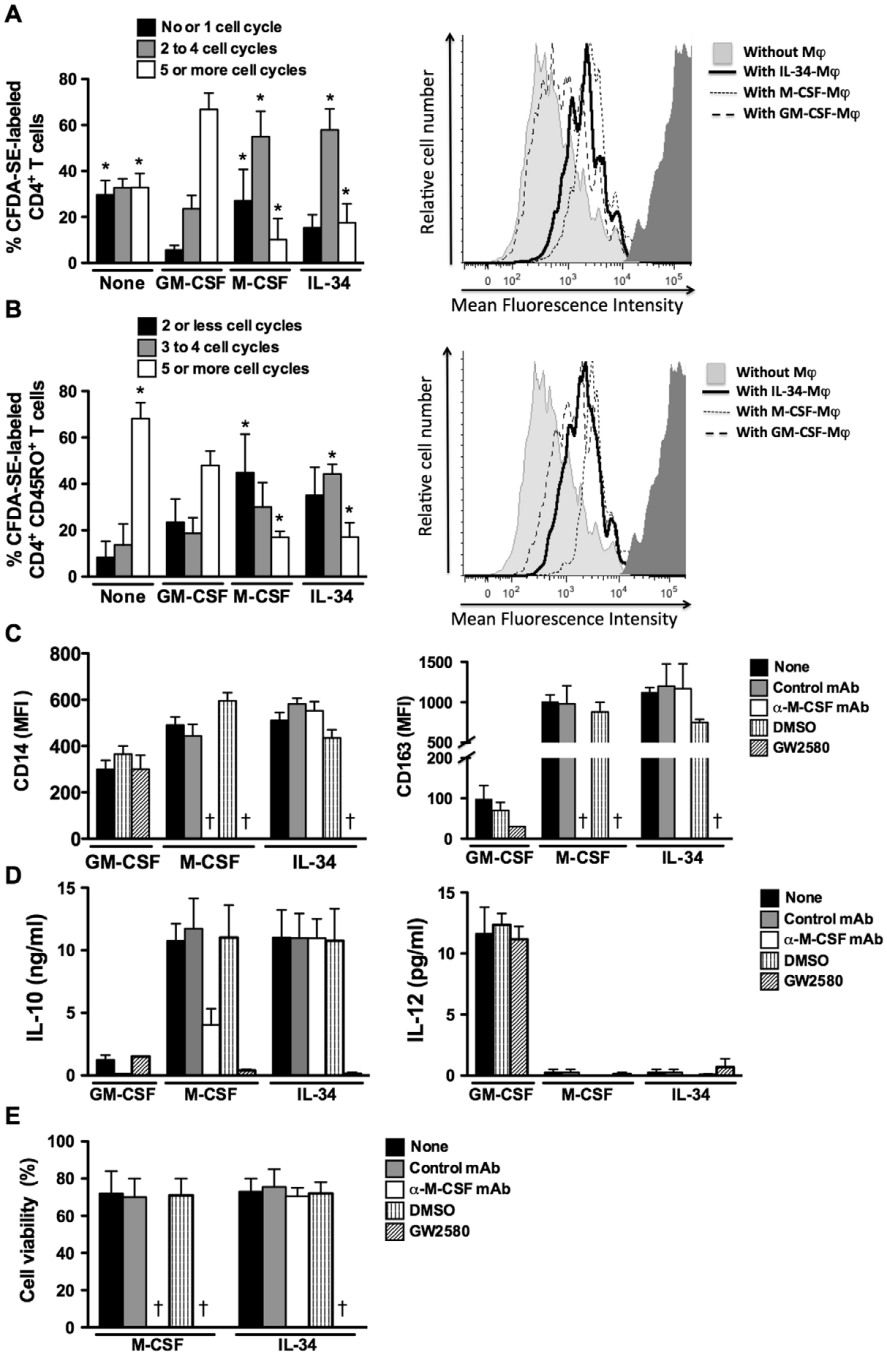
IL-34 induces the generation of immunosuppressive M2 independently of M-CSF. *A*&*B, IL-34-Mφ are immunosuppressive.* LPS-stimulated IL-34-Mφ, M-CSF-Mφ and GM-CSF-Mφ were cultured with CFDA-SE labeled CD4^+^ T cells in MLR experiments (*A*) or with CFDA-SE labeled memory CD45RO^+^ T cells in T cell proliferation assays (*B*); results are expressed as a percentage of cells depending on the number of cycle division (mean ± SD, n = 4). * p<0.05, compared to GM-CSF-Mφ. Right panels, representative histograms of CFDA-SE labeling from one donor. *C*&*D, IL-34 switches monocytes into M2 in an M-CSF-independent manner.* Analysis of the expression of CD14 and CD163 (*C*), and of the production of IL-10 and IL-12 (quantified after a 24 h stimulation with LPS) (*D*) by IL-34-Mφ, M-CSF-Mφ and GM-CSF-Mφ generated in the absence or presence of a neutralizing anti-M-CSF mAb, an isotype control mAb, the kinase inhibitor GW2580, or the drug diluent. Results are expressed in MFI values or in ng/ml (mean ± SD, n = 4). *E, Analysis of cell viability.* The viability of monocytes cultured with IL-34, M-CSF or GM-CSF, in the absence of presence of a neutralizing anti-M-CSF mAb, an isotype control mAb, the kinase inhibitor GW2580, or the drug diluent, was determined at day 3 by annexinV labeling. Results are expressed as a percentage of living cells (mean ± SD, n = 4). † means >90% of died cells.

Macrophages participate to the control of T cell responses [4], [6], [10]. We have thus evaluated the ability of IL-34-Mφ to affect T cell proliferation. In agreement with studies showing that IL-10^high^ IL-12^low^ M2 are immunosuppressive [4], we observed, in mixed lymphocyte reaction (MLR) assays, that IL-34-Mφ exhibitd lower T cell costimulatory properties than GM-CSF-Mφ (Fig. 4A). Moreover, IL-34-Mφ suppressed TCR-dependent T cell proliferation more efficiently than GM-CSF-Mφ (Fig. 4B), showing that they present potent immunosuppressive properties.

These results show that IL-34 drives the differentiation of monocytes into immunosuppressive Mφ.

### IL-34 induces M2 via CD115 and independently of M-CSF

M-CSF is produced by monocytes and induces their differentiation into M-CSF-Mφ by acting via CD115 [10], [32]. We therefore evaluated whether the ability of IL-34 to induce monocyte differentiation into M2 may also occur through autocrine secretion of M-CSF. A neutralizing anti-M-CSF mAb did not affect the ability of IL-34 to induce the generation of IL-34-Mφ, as evidenced by the maintenance of CD14 expression (Fig. 4C, left panel), the increase of CD163 expression (Fig. 4C, right panel) and the acquisition of an IL-10^high^/IL-12^low^ phenotype (Fig. 4D). The expression of CD80 and CD86 by IL-34-Mφ was not modulated by an anti-M-CSF mAb (data not shown). In parallel, we analyzed the expression of M-CSF during the course of monocyte differentiation into IL-34-Mφ. No M-CSF was detected in the culture supernatants collected from D1 to D5 and the expression of the transcript encoding M-CSF was not modulated by IL-34 during the 5-days culture (data not shown). In contrast, monocytes exposed to M-CSF plus an anti-M-CSF mAb rapidly died in culture (Fig. 4C&D), a result in agreement with the fact that M-CSF is a survival factor for monocytes/macrophages.

In agreement with the fact that IL-34 and M-CSF signal via CD115, the c-fms tyrosine kinase inhibitor GW2580 [12] impaired the survival of monocytes cultured with IL-34 or M-CSF (Fig. 4C–D). Finally, we observed that the viability of cells cultured with IL-34 and M-CSF was similar from D1 to D5 (Fig. 4E and data not shown).

Collectively, these results demonstrate that IL-34 switches monocytes into M2 by acting through CD115, in an M-CSF-independent manner.

### GM-CSF and IFNγ prevent IL-34-Mφ generation

**Figure 5:**
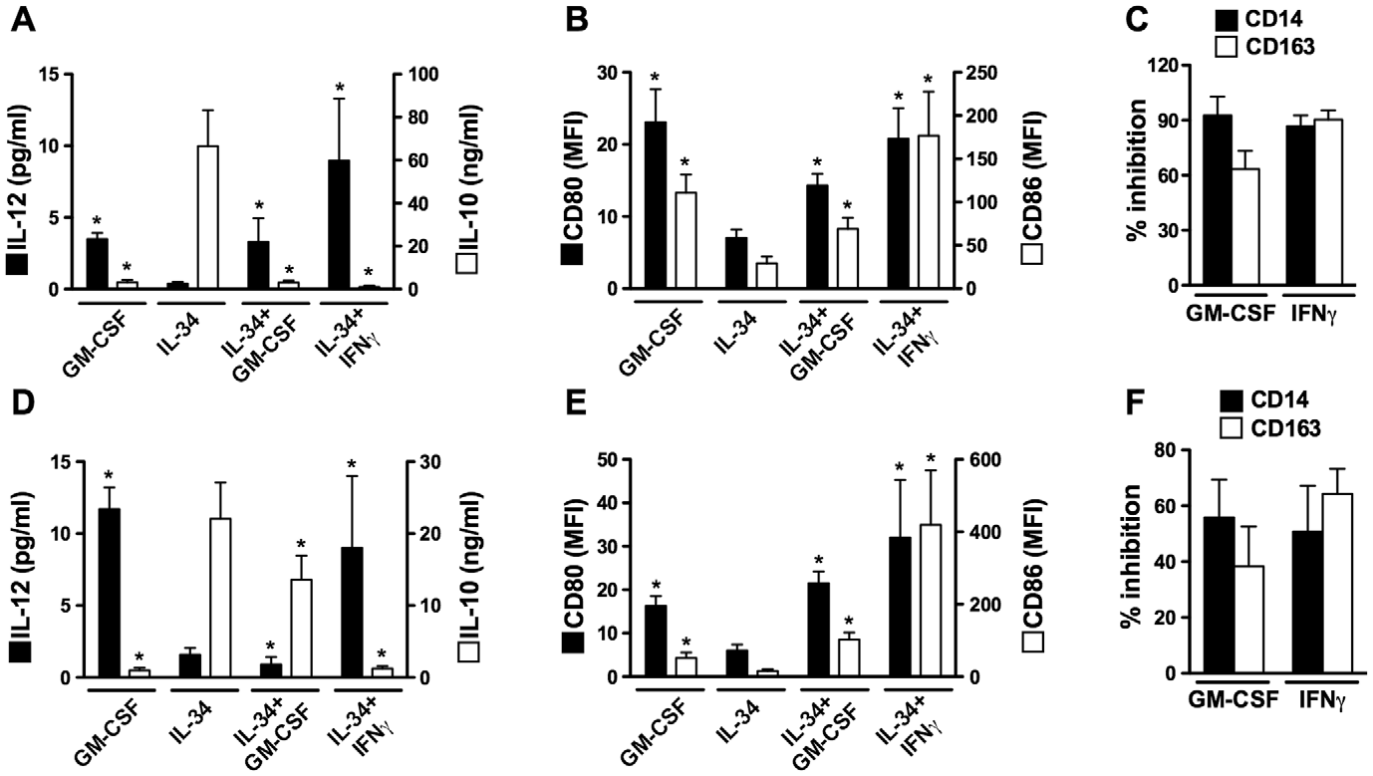
GM-CSF and IFNγ prevent the generation of IL-34-Mφ. *A–C, GM-CSF and IFNγ prevent the differentiation of monocytes into immunosuppressive IL-34-Mφ.* IL-34-Mφ and GM-CSF-Mφ were generated in the absence or presence of GM-CSF or IFNγ before phenotype analysis. *D–F, IFNγ skews monocyte differentiation from IL-34-Mφ into immunostimulatory Mφ.* IL-34-Mφ and GM-CSF-Mφ were cultured for 3 days in the absence or presence of GM-CSF or IFNγ before phenotype analysis. The expression of CD14 and CD163 (*C*&*F*) was analyzed on non-stimulated cells. The production of IL-10 and IL-12 (*A*&*D*) and the expression of CD80 and CD86 (*B*&*E*) was analyzed after LPS stimulation. Results are expressed in MFI values, in pg/ml (IL-12) or ng/ml (IL-10), or as a percentage of inhibition (mean ± SD, n = 7), (*A*,*B*,*D*&*E*), * p<0.05, compared to IL-34-Mφ.

While immunosuppressive M2 play a central role in immune homeostasis, their local accumulation is detrimental in some pathological situations, such as in cancer [4], [5], [33]. We thereby screened a panel of cytokines (IL-1β, IL-2, IL-3, IL-4, IL-5, IL-7, IL-8, IL-9, IL-10, IL-12, IL-15, IL-17A, IL-17E, IL-19, IL-20, IL-21, IL-22, IL-23, IL-24, IL-26, IL-29, TNFα, GM-CSF, OSM, or IFNγ) for their ability to prevent the generation of IL-34-Mφ. Monocytes were incubated with the cytokines tested, in the presence of IL-34, and the phenotype of the cells was analyzed after 5 days, with a focus on the production of IL-10 and IL-12 and on the expression of CD80 and CD86. Among all the cytokines tested, GM-CSF and IFNγ were the most potent in preventing IL-34-Mφ generation (Fig. S1). More precisely, compared to IL-34-Mφ, monocytes cultured with IL-34 plus GM-CSF or IFNγ retained the capacity to produce IL-12 (Fig. 5A) and to express high levels of CD80 and CD86 in response to LPS (Fig. 5B), while producing undetectable levels of IL-10 (Fig. 5A). Moreover, monocytes cultured with IL-34 plus GM-CSF or IFNγ expressed lower levels of CD14 and CD163 than IL-34-Mφ (Fig. 5C). The ability of GM-CSF and IFNγ to prevent the generation of IL-34-Mφ was dose-dependent, significant at 2 or 20 ng/ml, depending on the parameters analyzed, and maximal at the highest concentration tested (50 ng/ml) (Fig. S2).

These results show that GM-CSF and IFNγ counteract the ability of IL-34 to generate immunosuppressive Mφ.

### IFNγ skews established IL-34-Mφ cells from an immunosuppressive to an immunostimulatory IL-12^high^ IL-10^low^ phenotype

Macrophages are characterized by their plasticity, and dynamic changes in Mφ polarization may occur in some pathologies [4]. We previously showed that cytokines that prevent the generation of M-CSF-Mφ may also induce their reversion into immunostimulatory M1 [34]. We thus evaluated whether GM-CSF and IFNγ may also revert the phenotype of established IL-34-Mφ. Upon IFNγ exposure, IL-34-Mφ recovered the capacity to produce high levels of IL-12 (Fig. 5D), and to express CD80 and CD86 in response to LPS (Fig. 5E), while they expressed lower levels of IL-10 (Fig. 5D), CD14 and CD163 (Fig. 5F) than IL-34-Mφ. IL-34-Mφ, exposed to GM-CSF for 3 days, recovered the ability to express CD80 and CD86 in response to LPS (Fig. 5E) but remained unable to produce IL-12 (Fig. 5D). The ability of GM-CSF and IFNγ to modulate the expression of these markers was dose-dependent, significant at 2 or 20 ng/ml, depending on the parameters analyzed, and maximal at the highest concentration tested (50 ng/ml) (Fig. S3).

In addition to confirming the plasticity of Mφ [6], [34], these results show that IFNγ switches established IL-34-Mφ cells into Mφ with an immunostimulatory phenotype.

## Discussion

IL-34 signals via M-CSF-R and promotes human monocyte survival [12], [13], [17]. Different observations suggest that IL-34 and M-CSF might have non-redundant and complementary roles [13], [17], [18], [19], [20]. As Mφ participate to the control of inflammation, tissue homeostasis and metabolism, to identify the role of IL-34 on monocyte differentiation may have potential therapeutic applications, especially when the relative expression of IL-34 and M-CSF in pathological situations will be established. To date, the phenotype of the human myeloid cells exposed to IL-34 remains unexplored. We show here that (i) IL-34 gives rise to immunosuppressive Mφ with a phenotype similar to the one induced by M-CSF, (ii) that GM-CSF and IFNγ prevent their generation and, (iii) that IFNγ skews established IL-34-Mφ into immunostimulatory IL-12^high^ IL-10^low^ M1. Previous studies have reported that M1 and M2 exhibit different profiles of chemokines and chemokine receptors [29], [30], [31]. We observed that IL-34-Mφ and M-CSF-Mφ are indistinguishable, based on selected chemokines and chemokine receptors. Interestingly, GM-CSF-Mφ express higher levels of the chemokines CCL1, CCL17, CCL22, CCL24 and CX3CL1, and of the chemokine receptors CCR2 and CXCR1, than IL-34-Mφ and M-CSF-Mφ. These molecules may constitute, in addition to the reliable markers IL-10 and IL-12, a molecular signature allowing discriminating M1 and M2.

Although it has been suggested that M-CSF and IL-34 may differentially modulate the production of some cytokines by monocytes [13], our results show that the effects of IL-34 and M-CSF on monocyte differentiation (in term of phenotypes and functions) are superimposable, a result consistent with data from others showing that human IL-34 and M-CSF are equivalent in inducing the proliferation of a mouse macrophage cell line transfected with the human M-CSF-R [17]. Our results also support a previous study showing that IL-34 was as effective as M-CSF in inducing the survival/proliferation of human monocytes and the formation of CFU-M [12]. Although our results do not allow excluding the existence of another receptor for IL-34, distinct from M-CSF-R, as previously suspected [17], [18], we demonstrate, in agreement with others [12], [13], that IL-34 acts on human monocytes via the M-CSF-R. Moreover, we also report that IL-34 acts on monocytes independently of endogenous M-CSF. Reciprocally, although human monocytes express IL-34 mRNA, we failed in detecting IL-34 secretion by monocytes in response to M-CSF, suggesting that M-CSF may also act independently of endogenous IL-34. Finally, in support of our results showing that IL-34 mimics the activity of M-CSF on the differentiation of human monocytes, it has been reported by others that IL-34 can replace M-CSF for osteoclast generation [21], [22].

IL-34 and M-CSF mRNA are expressed in different tissues and differences in their spatiotemporal expression have been reported [17], [18], [19], [20]. Moreover, no compensatory IL-34 mRNA expression has been observed in M-CSF-deficient mice [17]. Both these observations suggested that IL-34 and M-CSF have complementary and independent roles. More precisely, IL-34 mRNA levels are higher than M-CSF mRNA in skin, salivary glands, and in most areas of the brain [17], [35], [36]. Based on our results, it is tempting to speculate that, in these organs, IL-34, rather than M-CSF, may contribute to the generation of immunosuppressive M2, able to tune inflammation and to promote tissue repair. Interestingly, microglia and bone marrow-derived Mφ are tightly down-regulated to avoid excessive and detrimental immune responses. Our results suggest that IL-34, expressed by neurons and meningeal cells [37], may contribute to the generation and/or to the maintenance of the phenotype of brain-resident myeloid cells.

We report that IL-34 induces the generation of Mφ which a phenotype and functions similar to the ones of human tumor-associated macrophages (TAM) [10]. TAM accumulate in various solid tumors, and, in most of them, their density is correlated with poor prognostic [4], [5], [10]. In addition to immunosuppressive properties, TAM are trophic for tumors and favor angiogenesis, dissolution of connective tissues and metastasis [4], [5], [6], [10]. In additional experiments, we have also observed that IL-34-Mφ produce similar levels of TGFβ, MMP9, and VEGF than M-CSF-Mφ (personal data). TAM derive from circulating monocytes that differentiate locally into M2 in response to tumor microenvironmental factors, such as M-CSF and IL-6 [10]. Recently, DeNardo and coll reported that, in breast cancer, some antitumoral drugs induce the production of both IL-34 and M-CSF by mammary epithelial cells, suggesting that, in some circumstances, therapeutic strategies may favor tumor immune escape [25]. Based on these observations, it appears crucial to analyze, in various tumors, the relative expression of M-CSF and IL-34 by tumoral and tumor-associated stromal cells, in order to determine whether IL-34 could participate to tumor-induced immunosuppression and to determine the relative roles of IL-34 and M-CSF in different types of tumors.

While M2 maintain tissue homeostasis and prevent excessive immune responses, their accumulation in some pathologies, such as in tumor, is detrimental. Strategies to prevent cell generation and/or to reverse the immunosuppressive properties of M2 may increase the efficacy of anti-tumor immunotherapies. Interestingly, we observed that IFNγ (i) switches established IL-34-Mφ into immunostimulatory M1 and, (ii) prevents the generation of IL-34-Mφ. IFNγ is a potent activator of myeloid cells inducing the generation of immunostimulatory M1 and, as previously observed, preventing the generation of TAM-like in ovarian cancer [34]. In addition, GM-CSF was able to prevent IL-34-Mφ generation. Recently, by analyzing the transcriptional profiles of GM-CSF- and M-CSF-induced Mφ, it has been observed that the expression of the M-CSF-induced genes was counteracted by the addition of a low dose (1 ng/ml) of GM-CSF [38]. We also observed that GM-CSF (used at 1 to 20 ng/ml), prevented the generation of M-CSF-Mφ (personal unpublished data). Supporting these observations, GM-CSF suppresses, in a murine myeloid cell line, the expression of the M-CSF-R mRNA [32] through the transcriptional activation of a ribonuclease degradation system [27]. In contrast, IFNγ does not modulate M-CSF-R mRNA expression by monocytes [39]. As IL-34 signals via M-CSF-R, this result may explain why GM-CSF prevents the effect of IL-34 on monocytes while GM-CSF appears less potent than IFNγ in reverting the immunosuppressive phenotype of IL-34-Mφ, although we cannot exclude that this could be achieved after a prolonged time of exposure.

In conclusion, our results identify IL-34 as a novel factor involved in the control of Mφ polarization. By acting through the M-CSF-R, IL-34 switches monocytes into immunosuppressive Mφ. IL-34 may thereby play a central role in immune homeostasis, especially in the brain and skin where IL-34 is constitutively expressed. Our results also suggest that, in order to reverse tumor-associated immunosuppression mediated by myeloid cells, strategies based on the inhibition of M-CSF-R should be privileged to prevent the effects of both IL-34 and M-CSF.

## Supporting Information

Text S1 **Supporting materials and methods.**
(DOC)

Figure S1 **Analysis of the ability of cytokines to prevent the generation of IL-34-Mφ.** Monocytes were cultured for 5 days with IL-34, in the absence or presence of IL-1β, IL-2, IL-3, IL-4, IL-5, IL-7, IL-8, IL-9, IL-10, IL-12, IL-15, IL-17A, IL-17E, IL-19, IL-20, IL-21, IL-22, IL-23, IL-24, IL-26, IL-29, OSM, GM-CSF, IFNγ, or TNFα. The expression of CD80 and CD86 (*A*) and the production of IL-10 and IL-12 (*B*) were determined after 48 h stimulation with 200 ng/ml LPS. Results were compared to those from GM-CSF-Mφ and M-CSF-Mφ. Results are expressed in MFI values or in pg/ml (IL-12) or ng/ml (IL-10). Results are representative of one of three experiments.
(TIF)

Figure S2 **Dose-dependent analysis of the inhibitory activity of IFNγ and GM-CSF on macrophage polarization.** IL-34-Mφ, M-CSF-Mφ and GM-CSF-Mφ were generated in the absence or presence of 2, 10 or 50 ng/ml GM-CSF or IFNγ. The expression of CD163 (*A*) was analyzed on non stimulated cells; the expression of CD80 (*B*) and CD86 (*C*) and the production of IL-12 and IL-10 (*D*) were analyzed after 48 h stimulation with 200 ng/ml LPS. Results are expressed in MFI values or in pg/ml (IL-12) or ng/ml (IL-10) (mean ± SD, n = 4).
(TIF)

Figure S3 **Dose-dependent analysis of the inhibitory activity of IFNγ and GM-CSF on macrophage reversion.** IL-34-Mφ, M-CSF-Mφ and GM-CSF-Mφ were cultured for 3 days in the absence or presence of 2, 10 or 50 ng/ml GM-CSF or IFNγ. The expression of CD80 and CD86 (*A*) and the production of IL-12 and IL-10 (*B*) were analyzed after 48 h stimulation with 200 ng/ml LPS; results are expressed in MFI values or in pg/ml (IL-12) or ng/ml (IL-10) (mean ± SD, n = 4).
(TIF)
