# Correction: IL-34 Induces the Differentiation of Human Monocytes into Immunosuppressive Macrophages. Antagonistic Effects of GM-CSF and IFNγ

**DOI:** 10.1371/annotation/a732b069-ac19-4ca4-8474-97cbe0a7d7ee

**Published:** 2014-01-06

**Authors:** Etienne D. Foucher, Simon Blanchard, Laurence Preisser, Erwan Garo, Norbert Ifrah, Philippe Guardiola, Yves Delneste, Pascale Jeannin

Affiliation number 1 for the first, second, third, fourth, seventh and eighth authors is incorrect. The correct affiliation is: LUNAM Université, Université d'Angers, Equipe Labellisée Ligue Nationale contre le Cancer, Angers, France. 

