# Supplementary material for: IL-34 Induces the Differentiation of Human Monocytes into Immunosuppressive Macrophages. Antagonistic Effects of GM-CSF and IFNγ
Source: PLoS One. 2013 Feb 8;8(2):e56045. doi: 10.1371/journal.pone.0056045 (PMC3568045; doi:10.1371/journal.pone.0056045)
Supplement: Text S1 — Supporting materials and methods. (DOC) [file pone.0056045.s001.doc]

**Supporting information**

**Supporting Materials and Methods**

**Role of cytokines on the generation of IL-34-Mϕ**. Monocytes were cultured for 5 days with 20 ng/ml GM-CSF (GM-CSF-Mϕ), 50 ng/ml M-CSF (M-CSF-Mϕ) or 50 ng/ml IL-34 (IL-34-Mϕ), in the absence or presence of 50 ng/ml of the following cytokines: IL-1β, IL-2, OSM (Miltenyi Biotec, Bergisch Gladbach, Germany), IL-4, GM-CSF (CellGenix, Freiburg, Germany), IL-3, IL-7, IL-8, IL-9, IL-12, IL-15, IL-21, IFNγ (Immunotools, Friesoythe, Germany), IL-5, IL-17A, IL-17E, IL-19, IL-20, IL-22, IL-23, IL-24, IL-26, IL-29, or TNFα (R&D Systems, Abingdon, United Kingdom). The expression of CD80 and CD86 (analyzed by flow cytometry) and the production of IL-10 and IL-12 (quantified by ELISA) were analyzed after 2 days activation with 200 ng/ml LPS. Results are expressed in MFI values, in pg/ml (IL-12) or in ng/ml (IL-10). Results are representative of one out of three experiments.

**Dose-dependent inhibition and reversion of macrophage polarization**. Monocytes were cultured for 5 days with 50 ng/ml M-CSF (M-CSF-Mϕ) or IL-34 (IL-34-Mϕ) or with 20 ng/ml GM-CSF (GM-CSF-Mϕ). In inhibition experiments, 2, 20 or 50 ng/ml GM-CSF or IFNγ were added at day 0. The expression of CD163 was analyzed on non stimulated cells and the expression of CD80 and CD86 and the production of IL-10 and IL-12 were analyzed after 48 h stimulation with 200 ng/ml LPS. In reversion experiments, day-5 IL-34-Mϕ and M-CSF-Mϕ were incubated with 2, 20 and 50 ng/ml GM-CSF or IFNγ for 3 days. The expression of CD86 and the production of IL-10 and IL-12 were analyzed after 48 h stimulation with 200 ng/ml LPS. Results are expressed in MFI values, in pg/ml (IL-12) or in ng/ml (IL-10) (mean ± SD, n=4).
