# Supplementary figures and images for: IL-34 Induces the Differentiation of Human Monocytes into Immunosuppressive Macrophages. Antagonistic Effects of GM-CSF and IFNγ
Source: PLoS One. 2013 Feb 8;8(2):e56045. doi: 10.1371/journal.pone.0056045 (PMC3568045; doi:10.1371/journal.pone.0056045)

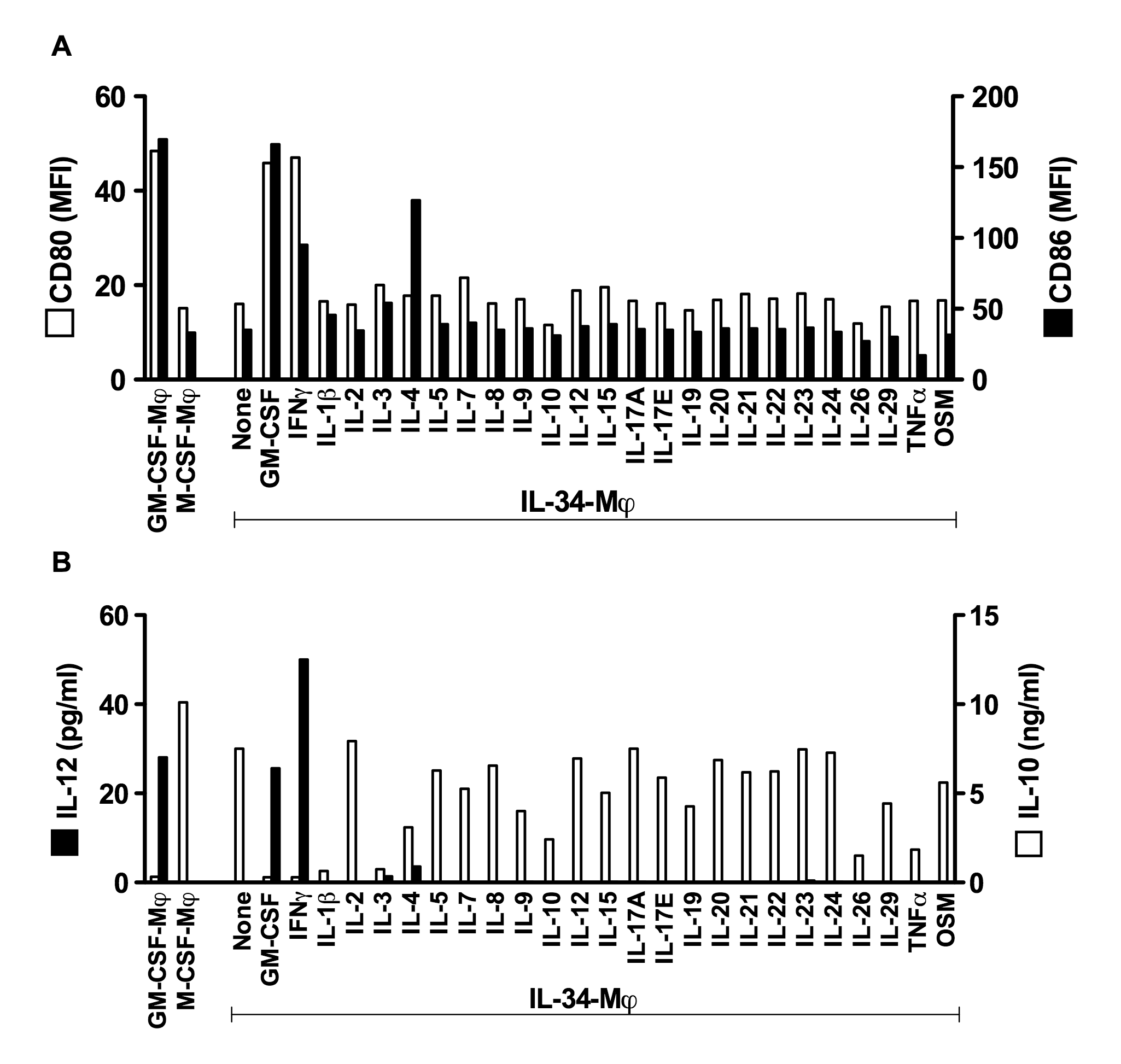

Supplement: Figure S1 — Analysis of the ability of cytokines to prevent the generation of IL-34-Mφ. Monocytes were cultured for 5 days with IL-34, in the absence or presence of IL-1β, IL-2, IL-3, IL-4, IL-5, IL-7, IL-8, IL-9, IL-10, IL-12, IL-15, IL-17A, IL-17E, IL-19, IL-20, IL-21, IL-22, IL-23, IL-24, IL-26, IL-29, OSM, GM-CSF, IFNγ, or TNFα. The expression of CD80 and CD86 (A) and the production of IL-10 and IL-12 (B) were determined after 48 h stimulation with 200 ng/ml LPS. Results were compared to those from GM-CSF-Mφ and M-CSF-Mφ. Results are expressed in MFI values or in pg/ml (IL-12) or ng/ml (IL-10). Results are representative of one of three experiments. (TIF) [file pone.0056045.s002.tif]

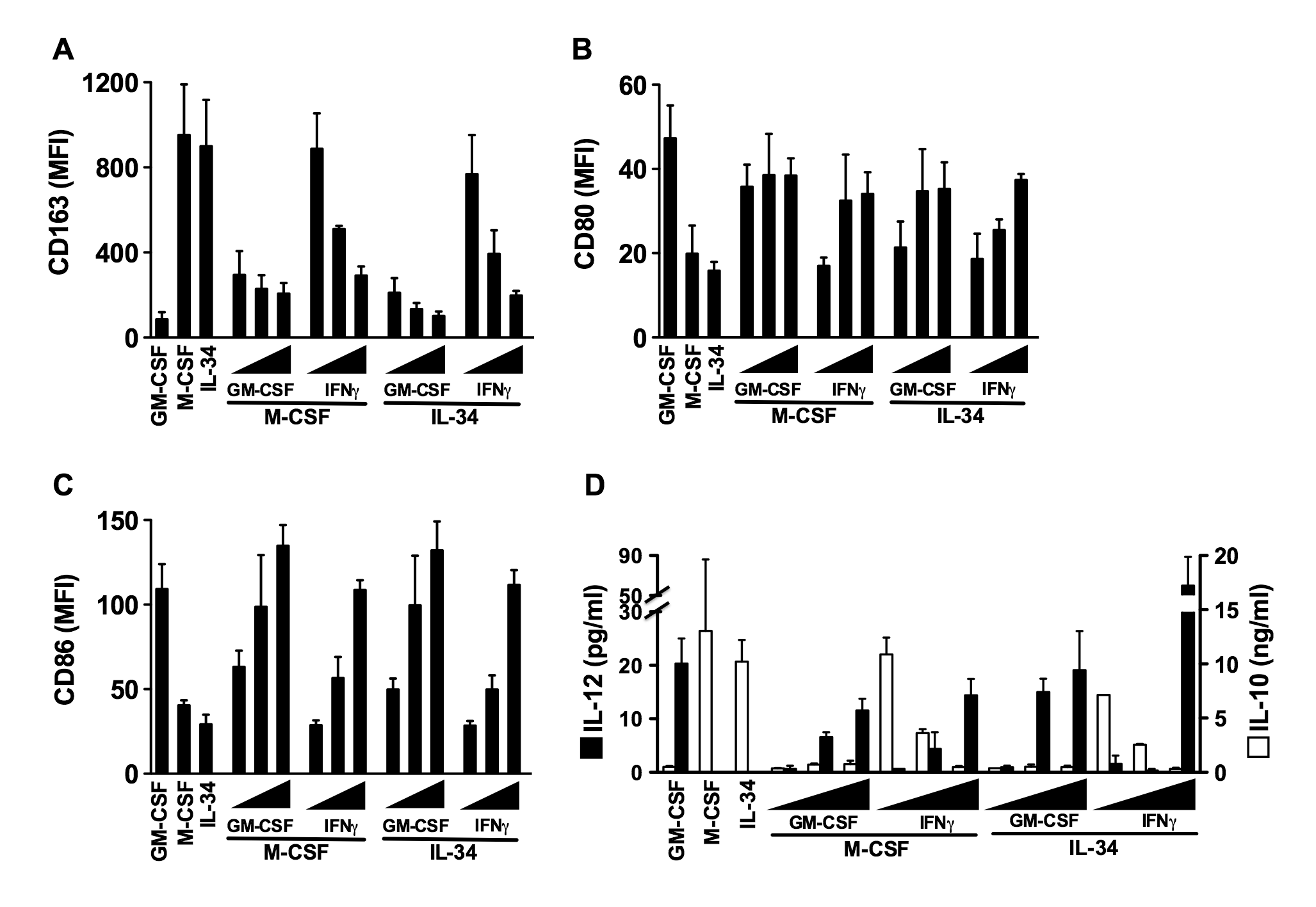

Supplement: Figure S2 — Dose-dependent analysis of the inhibitory activity of IFNγ and GM-CSF on macrophage polarization. IL-34-Mφ, M-CSF-Mφ and GM-CSF-Mφ were generated in the absence or presence of 2, 10 or 50 ng/ml GM-CSF or IFNγ. The expression of CD163 (A) was analyzed on non stimulated cells; the expression of CD80 (B) and CD86 (C) and the production of IL-12 and IL-10 (D) were analyzed after 48 h stimulation with 200 ng/ml LPS. Results are expressed in MFI values or in pg/ml (IL-12) or ng/ml (IL-10) (mean ± SD, n = 4). (TIF) [file pone.0056045.s003.tif]

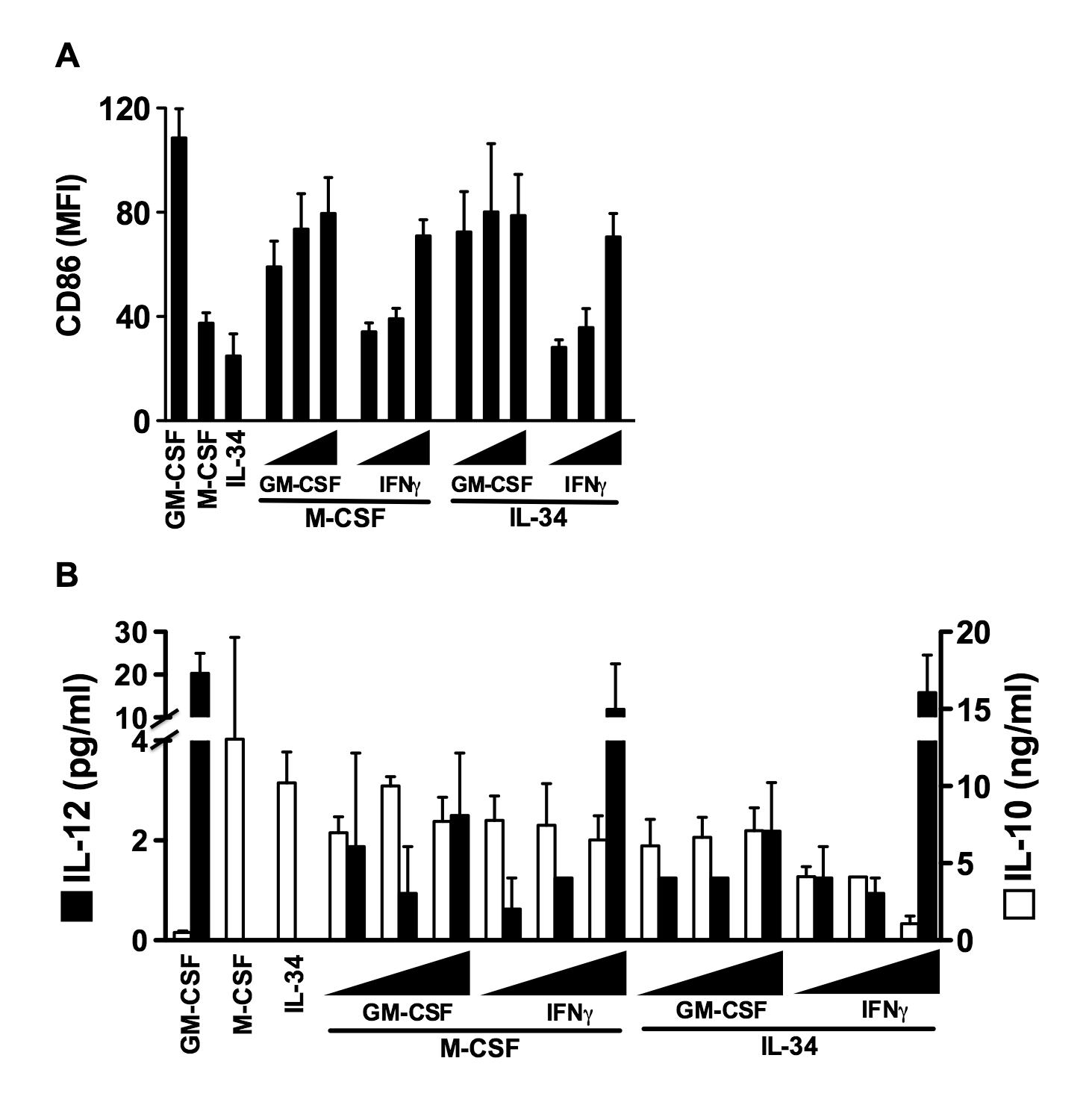

Supplement: Figure S3 — Dose-dependent analysis of the inhibitory activity of IFNγ and GM-CSF on macrophage reversion. IL-34-Mφ, M-CSF-Mφ and GM-CSF-Mφ were cultured for 3 days in the absence or presence of 2, 10 or 50 ng/ml GM-CSF or IFNγ. The expression of CD80 and CD86 (A) and the production of IL-12 and IL-10 (B) were analyzed after 48 h stimulation with 200 ng/ml LPS; results are expressed in MFI values or in pg/ml (IL-12) or ng/ml (IL-10) (mean ± SD, n = 4). (TIF) [file pone.0056045.s004.tif]
